# Factors associated with sexual violence among female administrative staff of Mekelle University, North Ethiopia

**DOI:** 10.1186/s13104-019-4860-5

**Published:** 2020-01-07

**Authors:** Sara Bahta Galu, Haftu Berhe Gebru, Yohannes Tesfay Abebe, Kahsu Gebrekirstos Gebrekidan, Atsede Fantahun Aregay, Kidane Gebremicheal Hailu, Gerezgiher Buruh Abera

**Affiliations:** 1 Ayder Comprehensive Specialized Hospital, Mekelle, Ethiopia; 2 College of Health Science, School of Nursing, Mekelle University, Mekelle, Ethiopia; 3 Dr Tewelde Legesse Health Science College, Mekelle, Ethiopia

**Keywords:** Sexual violence, Female, Administrative staff, Mekelle University

## Abstract

**Objective:**

To assess factors associated with sexual violence among female administrative staffs of Mekelle University, North Ethiopia.

**Results:**

From the total number of participants, 188 (52.8%) had shift work and 110 (30.9%) of these had day and night shift. About half 180 (50.2%) of the participants face sexual violence similarly, 53 (14.9%) of the victims of violence performed by their boss. In multiple logistic regression analysis young age [AOR: 2.319 (1.059–5.075)], educational status of secondary school or less [AOR: 1.981 (1.126–3.485)], office and students related workplace [AOR: 4.143 (1.975–8.687), 2.887 (1.396–5.973)], having night shift [AOR: 2.131 (1.258–3.611)], having multiple partner (AOR: 8.916 (3.052–26.047)] and knowing other female violated in office [AOR: 3.920 (2.326–6.606)] were the factors associated with sexual violence.

## Introduction

As defined by the World Health Organization (WHO) sexual violence is any sexual act, attempt to a sexual act, unwanted sexual comments or advances, or acts to traffic directed against a person’s sexuality using force, by any person regardless of their relationship to the victim, and in any setting, such as home and work [1]. Sexual violence is a severe public health problem that has drawn global attention as a worldwide problem [2]. Sexual violence results in short and long-term adverse psychological and social outcomes. In addition, it has significant reproductive health implications such as increased risk for sexually transmitted infections and unwanted pregnancies [1, 3]. Though women and girls suffer disproportionately, sexual violence can occur to anybody at any age. It is also an act of violence that can be perpetrated by parents, caregivers, acquaintances, strangers, and intimate partners [4].

Globally 1 in 3 (35%) of women have experienced either physical and/or sexual intimate partner violence or non-partner sexual violence in their lifetime [5]. Based on a report from the WHO, the rate of violence against women in African countries was estimated to be 36.3% [6]. Sexual violence is a widespread problem in sub-Saharan Africa [7]. In Ethiopia, like the other sub-Saharan Africa countries the rate of sexual violence is high which is 59% of ever-partnered women experienced sexual violence [8]. As part of Ethiopia, in Tigray, sexual violence is still high, for example, a research done in Adigrat hospital shows 60.2% of rape cases occurred among children and adolescents [9].

Millions faced violence and harassment in workplaces [10]. The potential perpetrators of sexual violence and harassment can be anyone, including employers, workers and third parties (clients, customers, service providers, users, patients) [11]. Moreover, victims can be targeted based on different factors such as gender, economic class, race, sexual orientation, disability, or a combination of them factors [10, 11]. Nevertheless, sexual violence and harassment affect women disproportionately, and men tend to be the perpetrators [11]. Accurately estimating the prevalence of sexual violence in the developing world is difficult because there are few studies done on sexual violence in employed women. Therefore, the aim of this study was to assess factors associated with sexual violence among female administrative staffs of Mekelle University, North Ethiopia.

## Main text

### Methods

This institution based cross-sectional study was conducted in Mekelle city, North Ethiopia which is 783 km far from Addis Ababa. Mekelle University has seven campuses, and colleges and all colleges were included in the study. The total number of female administrative staffs working in all campuses were 937. The study population was a sample of the female administrative staff at Mekelle University. The sample size was calculated using single population proportion formula. Using systematic sampling technique, 356 (sample size) females were selected using the Kith value of 3. Sexual violence was the dependent variable of the study whereas the independent variables were sociodemographic, substance-related, work status and psychosexual characteristics related variables.

Primary data was collected using a structured pre-tested and self-administered questionnaire that was developed from the existing literature [12, 13]. The questionnaire was first developed in English then translated to Tigrigna (local language). Before the actual time of data collection, a pre-test was conducted on 10% similar population in Mekelle city administration not included in the study. Data collectors were assigned to the seven campuses of Mekelle University to distribute and collect the questionnaire. Data entry was conducted using EPI-INFO version 7 computer software, and data cleaning and analysis was done using SPSS version 22. The data cleaning was performed by calculating frequency of each variable to check accuracy, inconsistency and missed the value of the data. Before analysis of the data, recoding of variables was conducted to make it easy for analysis. Descriptive statistics like frequency for normally distributed data and median with interquartile range for non-normally distributed data to all variables were computed. Bivariate logistic regression was used as method of analysis, variables statistically significant with P < 0.3 was taken to the multivariable logistic regression. For all statistical significance tests between each independent and dependent variable, significant at P < 0.05 was be considered reliable for the analysis of the study.

Sexual violence was operationalized as unwanted sexual act such as a verbal joke, question for sex, faced unwanted kiss, unwelcomed touching of woman’s body parts such as breast, genitalia, forced sexual attempt, forced sex without consent/will, forced sex that made a woman frightened and degrading in the past six months.

### Results

#### Sociodemographic characteristics

A total of 356 female administrative staff of Mekelle University participated in this study giving a response rate of 100%. From the total participants, 174 (48.9%) of them were in the age group 26–35 years, and 171 (48%) of the participants were married. Regarding ethnicity, majority (89%) of them were Tigrians as well as 270 (75.8%) females were Orthodox Christian. Two hundred thirty-four (65.7%) participants had educational status of certificate and above. About 22.2% of the participants were working as runners (transport patients from one department to other department in a hospital). Two hundred and fifteen (60.4%) of the participant females had a monthly salary of 500–1500 Birr (Table 1).

**Table 1:** Sexual violence vs sociodemographic characteristics of female employees of MU, 2018 (*n* = 356)

Variable	Sexual violence			
	Yes		No	
	Freq.	Percent	Freq.	Percent
Age in years				
15–25	39	11.0	50	14.0
26–35	96	27.0	78	21.9
36–45	42	11.8	46	12.9
> 45	3	0.8	2	0.5
Marital status				
Single	59	16.6	65	18.3
Married	80	22.5	91	25.6
Divorced	31	8.7	16	4.5
Widowed	10	2.8	4	1.1
Ethnicity				
Tigraway	160	44.9	157	44.1
Amhara	16	4.5	13	3.7
Others*	4	1.2	6	1.7
Religion				
Orthodox	123	34.6	147	41.3
Muslim	27	7.6	15	4.2
Others**	30	8.4	14	3.9
Educational status				
Primary education (1–8 grade)	25	7.0	15	2.8
Secondary education	47	13.2	35	9.8
Diploma and certificate	71	19.9	54	15.2
Degree and above	37	10.4	72	20.2
Residence				
Urban	180	50.2	176	49.4
Occupation				
Secretary	21	5.9	21	5.9
Receptionist	20	5.6	11	3.1
Runner	49	13.8	30	8.4
Library	19	5.3	18	5.1
Procter	32	9.0	29	8.1
Laundry	11	3.1	31	8.7
Registrar	19	5.3	17	4.8
Others****	9	2.5	19	5.3
Position at work				
Office manager	14	7.1	20	10.2
Coordinator	15	7.6	16	8.1
Customer officer	46	23.4	28	14.2
Department head	4	2.0	6	3.0
Others***	27	13.7	21	10.7
Monthly income in Birr				
500–1500	100	28.1	115	32.3
1501–2500	61	17.1	42	11.8
2501–3500	16	4.5	17	4.8
> 3500	3	0.8	2	0.6

*Afar, Erob; **Christian: Catholic, Protestant; ***immediate supervisors of different workplaces; ****cleaner, guard, administrative works such as accountant, human resource

#### Sexual violence

From the total number of participants, 188 (52.8%) had a shift on their work schedule and 110 (30.9%) of them had day and night shift. About half 180 (50.2%) of the participants face sexual violence. Similarly, 53 (14.9%) of the violence was performed by their boss. Workmates and students were also other common perpetrators. The number of female workers who face violence in their workplace was 122 (67.8%) whereas, the rest faced violence was happened outside their workplaces such as in bus and other places. Bullying was the common act of violence as reported by 82 (45.5%) of participants. One hundred fourteen (63.3%) of the victims didn’t know whether the perpetrators use a substance or not whereas from those who responded yes for substance use, 26 (57.8%) of the perpetrators used alcohol. From the total study participants, about 46.4% had no boyfriend and from those who had boyfriend 105 (29.5%) of them had regular sexual contact (Table 2).

**Table 2:** Sexual violence versus different variables among female administrative staffs of MU, 2018 (*n* = 356)

Variable	Sexual violence			
	Yes		No	
	Frequency	Percent	Frequency	Percent
Shift at work				
Yes	113	31.7	75	21.1
No	67	18.8	101	28.4
Program of the shift				
Day	28	7.8	20	5.6
Night	23	6.5	7	2.0
Day and night	62	17.4	48	13.5
Place where the act performed				
In Office	122	67.8		
Street	35	19.4		
Others*	23	12.8		
How the act was done				
Using force	45	25		
By kicking	10	5.5		
Bullying	82	45.5		
Verbal abusing	43	23.9		
Did the perpetrator use any substance				
Yes	45	25.		
No	21	11.6		
I don’t know	114	63.3		
Type of substance				
Alcohol	26	57.8		
Khat or others**	19	42.2		
History of substance violence				
Yes	101	56.1		
No	10	5.6		
I don’t know	69	38.3		
What type of substance				
Khat chewing	45	44.5		
Smoking	20	19.8		
Alcohol	27	26.7		
Others**	9	8.9		
Previous history of such practice of the perpetrator				
Yes	36	14.4		
No	21	11.7		
I don’t know	123	68.3		
Did the perpetrator has more than one partners				
Yes	85	47.2		
No	18	10		
I don’t know	77	42.8		
Do you know the perpetrator well				
Yes	138	76.7		
No	42	23.3		
Did the perpetrator have family				
Yes	95	68.8		
No	9	6.5		
I don’t know	34	24.6		
With whom did the perpetrator live				
Lonely	54	39.1		
Family	45	32.6		
I don’t know	39	28.3		
How old was the perpetrator				
Same as me	78	43.3		
Older than me	102	56.7		
Do you have a boyfriend				
Yes	107	30.1	58	16.3
No	73	20.5	118	33.1
If yes how often is your sexual relationship				
No sexual contact	26	7.3	19	5.3
Regular	66	18.5	39	10.9
Relationship other than your boyfriend				
Yes	41	11.5	5	1.4
No	66	18.5	53	14.9
With how many				
One	18	5.1	3	0.8
Two or more	23	6.5	2	0.6
History of sexual violence				
One time	84	46.6	5	2.8
Two times	24	13.3	4	2.2
Three times	32	17.8	0	0.0
More than four times	30	16.7	1	0.5
Do you know a woman who is violated in the workplace				
Yes	108	30.3	48	13.5
No	72	20.2	128	36.0

*Around workplace out of office; **drug, shisha

#### Factors associated with sexual violence

To assess the relationship between the dependent and independent variables, logistic regression analysis method was used. In binary logistic regression educational status, workplace, having a night shift, having boyfriend, sexual contact, having multiple partners and knowing other females violated in the office were statistically associated with sexual violence. Whereas, in multiple logistic regression the factors associated with sexual violence were: age, educational status, workplace, having a night shift, having multiple partners and knowing other female violated in office.

Females in the age group 15–25 years were two times at greater risk of sexual violence than the other age groups [AOR: 2.319 (1.059–5.075)]. Similarly, females in high school or lower with their educational status were approximately two times at greater risk of sexual violence as compared to their counterparts [AOR: 1.981 (1.126–3.485)]. Type of work was also strongly associated with sexual violence. As a result, females working in office related (secretary, runners) and student-related works (registrar, library, proctor) had 4 and 3 times chance of having sexual violence than the females working in other places (laundry and others) respectively [AOR: 4.143 (1.975–8.687), 2.887 (1.396–5.973)]. The participants who had night shifts were two times at greater risk than those who had not night shifts [AOR: 2.131 (1.258–3.611)]. Additionally, females having a history of multiple partners was approximately nine times more at risk of sexual violence as compared to their counterparts [AOR: 8.916 (3.052–26.047)]. Participants who know violence in their workplace were four times risky than those who did not [AOR: 3.920 (2.326–6.606)] (Table 3).

**Table 3:** Factors associated with sexual violence among the female administrative staff of MU, 2018 (*n* = 356)

Variable	Sexual violence		COR (95% C.I)	AOR (95% C.I)
	Yes	No		
Age in years				
15–25	39 (11%)	50 (14%)	0.634 (0.379–1.060)	2.319 (1.059–5.075)*
26–35	96 (26.9%)	78 (21.9%)	0.832 (0.464–1.492)	1.773 (0.950–3.311)
> 35	45 (16.6)	48 (13.5%)	1	1
Religion				
Christian	153 (43%)	161 (45.2)	0.528 (0.270–1.031)	0.565 (0.264–1.210)
Muslim	27 (7.6%)	15 (4.2%)	1	1
Educational status				
Secondary/lower	72 (20.2%)	48 (13.5%)	1.778 (1.138–2.777)*	1.981 (1.126–3.485)*
Higher education	108 (30.3%)	128 (36%)	1	1
Type of work				
Office related	90 (25.3%)	61 (17.1)	0.271 (0.147–0.500)*	4.143 (1.975–8.687)*
Student related	70 (19.7)	65 (18.2%)	0.371 (0.200–0.690)*	2.887 (1.396–5.973)*
Others	20(5.6%)	50 (14%)	1	1
Position at work				
Yes	104 (29.2%)	91 (25.6%)	1.278 (0.841–1.942)	1.288 (0.783–2.118)
No	76 (21.3%)	85 (23.9%)	1	1
Night shift				
Yes	85 (23.9)	55 (15.4%)	1.968 (1.277–3.034)*	2.131 (1.258–3.611)*
No	95 (26.7%)	121 (34%)	1	1
Boy friend				
Yes	107 (30.1%)	58 (16.3%)	2.982 (1.934–4.597)*	1.167 (0.549–2.481)
No	73 (20.5%)	118 (33.1%)	1	1
Sexual contact				
Yes	66 (18.5%)	39 (11%)	2.034 (1.274–3.245)*	1.103 (0.488–2.494)
No	114 (32%)	137 (38.5%)	1	1
Multiple partner				
Yes	41 (11.5%)	5 (1.4%)	10.088 (3.882–26.216)*	8.916 (3.052–26.047)*
No	139 (39%)	171 (48%)	1	1
Know violence in the workplace				
Yes	108 (30.3%)	48 (13.5%)	4.000 (2.561–6.249)*	3.920 (2.326–6.606)*
No	72 (20.2%)	128 (36%)	1	1

*COR* crude odds ratio, *AOR* adjusted odds ratio, *CI* confidence interval

*P < 0.05

### Discussion

This study aimed to assess sexual violence among female administrative staffs of Mekelle University. The magnitude of sexual violence in the workplace was 34.3%. Whereas in a study done to assess sexual harassment in workplace conducted in Lebanon, the prevalence of sexual violence was 41.9% [13]. In addition, this finding of the study was lower than a study done in Nepal that asses sexual violence in work place, which showed about 53.8% of women employees faced sexual violence in their workplace [14]. Similarly, a study done in Malaysia among employed women found that 52.7% sexual violence was conducted in workplace [15]. This variation might be due to differences in socioeconomic status, study participants, sample size and operational definition of the problem. In this study, for example, any action against the women was considered as sexual violence.

The current study revealed that the common perpetrators of sexual violence were bosses (14.9%), workmates (12.1%), students (9.3%), husbands (7%) and boyfriends (7.3%). Whereas, in a study conducted in Nigeria the perpetrators were clients and managers [13]. Similarly, in a study done in Denmark and Danish workplaces sexual violence was conducted by colleagues, supervisors, subordinates [16]. This difference is due to the time gap, study design, and a difference in sociodemographic status.

This study found that females working in student-related work such as registrar, proctor and library as well as females working in office related activities were at higher risk of sexual violence as compared to females working in others like laundry [AOR: 4.143 (1.975–8.687), 2.887 (1.396–5.973)]. This variation might be due to these areas require much interpersonal interaction that could make them vulnerable to violence. However, in a study done in Denmark and Danish workplaces, participants employed in care work were more often exposed to sexual violence by clients and customers than participants employed in private service, professional work, building and construction, and industrial work [16]. This variation might be due to a difference in the study area in which the study done in Denmark, and Danish workplaces were conducted in different areas. In developing countries, casual workers and informal sector workers appear to be particularly subject to harassment [16].

In a study done in Meda-Walabu, the associated factors of sexual violence were chat chewing, history of mother beaten by a partner and having regular boyfriend were positively associated with forced sex [17]. Additionally, A cross-sectional study in Iran showed that drugs used in their husbands were associated with sexual violence [18]. However, in this study substance use by the perpetrator and having boyfriend were not statistically associated with sexual violence. The variation in different studies is because this study was done in the workplace in which substance use is not allowed during working hours. Whereas the other studies were done in schools and the public.

In this study, the participants who had night shifts were two times at greater risk than those who had not night shifts [AOR: 2.131(1.258–3.611)]. This difference is because night time is a more convenient for violent people. Additionally, females having a history of multiple partners were approximately nine times more at risk of sexual violence as compared to their counterparts [AOR: 8.916 (3.052–26.047)]. Because in such situation disagreements can be apparent that leading to sexual/physical violence. However, it was difficult to compare these findings with other studies because there is no existing literature on this issue.

### Conclusion

This study found that sexual violence was committed against half of the female administrative staffs of Mekelle University. The typical perpetrators were bosses and workmates, and the common areas of violence were workplaces. The factors associated with sexual violence in this study were, younger age, high school and lower in educational status, office and students related workplace, having a night shift, having multiple partners and knowing other female violated in office.

## Limitations of the study

- Qualitative study is not included in the which was ideal to assess additional factors associated with sexual violence.
- Lack of published papers on sexual violence which make this study to compare with other studies.

## Data Availability

The datasets used and/or analyzed during the current study available from the corresponding author on reasonable request

## Abbreviations

AOR: adjusted odds ratio
CI: confidence interval
COR: crude odds ratio
WHO: World Health Organization
